# Association between skirt size and chronic liver disease in post-menopausal women: a prospective cohort study within the United Kingdom Trial of Ovarian Cancer Screening (UKCTOCS)

**DOI:** 10.1186/s12889-018-5308-x

**Published:** 2018-03-27

**Authors:** P. M. Trembling, S. Apostolidou, A. Gentry-Maharaj, J. Parkes, A. Ryan, S. Tanwar, M. Burnell, U. Menon, W. M. Rosenberg

**Affiliations:** 1 Institute for Liver and Digestive Health, Division of Medicine, University College London, Royal Free Hospital, Rowland Hill Street, NW3 2PF, London, UK; 2 Gynaecological Cancer Research Centre, Department of Women’s Cancer, Institute for Women’s Health, University College London, London, UK; 3 Public Health Sciences and Medical Statistics, Faculty of Medicine, University of Southampton, Southampton, UK

**Keywords:** Chronic liver disease, Cirrhosis, Skirt size, Body mass index, Obesity, UKCTOCS

## Abstract

**Background:**

We investigated the association between self-reported skirt size (SS) and change in SS, and incidence of chronic liver disease (CLD) in a prospective cohort study of women recruited to the UKCTOCS trial.

**Methods:**

Women recruited to UKCTOCS in England without documented CLD self-reported their current UK SS during trial participation and were asked to recall their SS when aged in 20s (via completion of a questionnaire 3–5 years after recruitment). Participants were followed up via electronic health record linkage and hazard ratios (HR) calculated for incident liver-related events (LRE).

**Results:**

Three hundred twenty-two (0.3%) of 94,124 women experienced a first LRE. Compared to SS ≤ 16, rates of LRE were higher in the SS ≥ 18 groups (both when aged in 20s and at questionnaire completion). Event rates were higher if there was no change in SS or an increase in SS, compared to a decrease in SS.

In the models adjusted for potential confounders, HRs for LRE were higher in the groups of women reporting SS ≥ 18 both when aged in 20s (HR = 1.39 (95% CI; 0.87–2.23)) and at questionnaire completion (HR = 1.37 (95% CI; 1.07–1.75)). Compared to a decrease in SS, HRs were higher in the no change (HR = 1.78 (95% CI; 0.95–3.34)) and increase (HR = 1.80 (95% CI; 1.01–3.21)) groups.

**Conclusion:**

CLD is associated with high SS and an increase in SS over time. These data suggest SS can be used in simple public health messages about communicating the risk of liver disease.

**Trial Registration:**

UKCTOCS is registered as an International Standard Randomised Controlled Trial, number ISRCTN22488978. Registered 06/04/2000.

**Electronic supplementary material:**

The online version of this article (10.1186/s12889-018-5308-x) contains supplementary material, which is available to authorized users.

## Background

Chronic liver disease (CLD) is a leading cause of death in the UK. It is estimated that 60,000 people in the UK have cirrhosis [1, 2] but over half of those affected are unaware of the diagnosis [3]. The main causes of CLD are alcohol, non-alcoholic fatty liver disease (NAFLD) and viral hepatitis.

NAFLD describes the process of hepatic accumulation of fat, ranging from benign steatosis, via liver inflammation (steatohepatitis) to progressive liver fibrosis and eventually cirrhosis, and can be considered the pathological manifestation in the liver of the metabolic syndrome. In addition to type 2 diabetes, hypertension and hypercholesterolaemia, high body mass index (BMI) is a significant driver for NAFLD, and is associated with increased risk of heart disease and stroke [4]. Although BMI is commonly used as a measure of body fat, it has been demonstrated that waist and hip measurements may be stronger predictors of body fat than BMI [5, 6].

We have previously demonstrated the association between increasing BMI and risk of CLD. In a large cohort of post-menopausal women we observed more clinical events attributable to cirrhosis amongst women who were overweight or obese compared to those with a normal BMI. Although there was no evidence of significant interaction between alcohol and BMI, the highest risk of liver disease was seen in women who were overweight or obese and consumed the most alcohol [7].

Skirt size (SS) could be an easily understood surrogate for BMI to communicate public health messages about the risks of obesity. Increase in self-reported SS in participants in the United Kingdom Collaborative Trial of Ovarian Cancer Screening (UKCTOCS) has been shown to be associated with increased breast cancer risk. A unit increase in UK SS (e.g. 12 to 14) every 10 years between 25 and postmenopausal age is associated with postmenopausal breast cancer risk of 33%. Validation of these results could provide women with a simple and easy to understand message, using SS [8]. We now explore the association between SS and change in SS and the incidence of CLD.

## Methods

### Study population

This prospective cohort study was nested in UKCTOCS, a UK-based randomised controlled trial investigating the effect of ovarian cancer screening on mortality. The trial design is described elsewhere [7–11]; briefly, between April 2001 and October 2005, post-menopausal women aged 50–74 in England, Wales and Northern Ireland were invited at random and 202,638 participants recruited to the trial. Participants were randomly allocated to one of three arms (no screening, annual serum CA125 measurement and then transvaginal ultrasound as a second line test, or ultrasound only). Recent data from the trial have demonstrated the predictive value of changes in CA125 levels to predict ovarian cancer [12], and reduced mortality in the multimodal arm [13].

UKCTOCS was approved by the UK North West Multicentre Research Ethics Committee (North West MREC 00/8/34). All women provided written consent. The current study was approved by the National Research Ethics Service (NRES) Committee London - Bentham (Ref: 05/Q0505/57) on 10th August 2011.

### Exposures

The exposures of interest in this study were BMI and SS of participants. At the time of recruitment, participants completed a questionnaire, which included self-reported height and weight. BMI was calculated (BMI (kg/m^2^) = weight (kg)/height (m)^2^) and categorised according to the World Health Organization’s definitions; normal (< 25 kg/m^2^), overweight (25- < 30 kg/m^2^) or obese (≥30 kg/m^2^). There were some extreme values in self-reported data and as there are no existing population estimates for the range of BMI we applied a pragmatic approach in order to include participants with biologically plausible BMI values. Therefore, participants were excluded if their reported height lay outside the range 140–210 cm, or their weight lay outside the range 25–200 kg, or where the calculated BMI was outside the range 16–65 kg/m^2^.

Participants were asked to complete a follow-up questionnaire 3–5 years post randomisation, and were asked to estimate their UK SS when they were in their early twenties and to report their current SS. Using the two SS responses overall change in SS and change in SS per year were calculated. In the UK SS range comprises of even numbers, for example in increase in SS from 12 to 14 is an increase in one UK SS.

### Categorisation of exposure variables

BMI was categorised according to World Health Organization classification as normal, overweight, or obese. SS was categorised using UK dress sizes as ≤16 and ≥ 18; the latter cut-off selected because of its association with an increased risk of cardiovascular morbidity [14]. The British Standards Institution defines UK size 16 as 100–104 cm, and size 18 as 105–109 cm, measured at the hips [15]. Change in SS was categorised as decrease, no change or increase in SS between when participants were in their early 20s and at their current age.

### Covariates

Participants reported, via the follow-up questionnaire, known comorbidities, comprising hypertension, heart disease, hypercholesterolaemia, stroke, diabetes, rheumatoid arthritis, osteoarthritis, osteoporosis (“do you have/are you being treated for any of the following conditions?”), and whether they currently smoked, all categorised as yes/no. Participants were asked “approximately how much alcohol on average do you drink each week, assuming one drink = a glass of wine, half a pint of lager or cider, a measure of spirits?” This was then categorised as none, < 1–15 units/week, 16–20 units/week and ≥ 21 units/week, assuming one drink is equivalent to one UK unit (10 ml or 8 g of pure alcohol) [16].

Participants were assigned a deprivation score using the Index of Multiple Deprivation 2007 (IMD) (continuous variable) [17], with a higher score indicating higher deprivation.

### Follow up

Participants in this study were followed through a ‘flagging’ study with NHS Digital which provided data on cancer registrations and deaths, with diagnosis and/or cause of death coded according to the International Classification of Diseases, version 10 (ICD-10). Hospital inpatient and outpatient data for 2001–10 were also available through linkage to the Hospital Episodes Statistics (HES) database. Each inpatient HES episode record reports a main diagnosis and up to 19 additional diagnoses. Outpatient records report a main diagnosis and up to 11 further diagnoses. Death records report the primary death code and additional diagnoses documented on the death certificate, comprising both ICD-10 codes and free text. Only participants in England were included in this study, due to availability of their relevant HES data. Participants entered the study at the point of return of the follow-up questionnaire, as this was the date that current comorbidities and SS data were ascertained. Women with pre-existing liver disease were excluded if a code of interest had been registered between recruitment to UKCTOCS and return of follow-up questionnaire.

### Outcome

First liver-related event (LRE) was deemed the main outcome measure. LRE was defined as a participant’s first presentation of a hospital admission, outpatient appointment or cancer registration with, or death from, a relevant ICD-10 code. These codes were K70 (alcoholic liver disease), K73 (chronic hepatitis) and K74 (fibrosis and cirrhosis) and are consistent with codes employed in other UK studies of cirrhosis [1, 18]. In addition K76 (other diseases of the liver, including fat) and codes related to decompensated liver disease (I85 (oesophageal varices), Z94.4 (liver transplant) and C22.0 (hepatocellular carcinoma)) were included. Death certificates were interrogated for ICD-10 codes of interest and free text relating to alcoholic liver disease or fatty liver.

### Statistical analysis

For the incidence analyses person-years of follow-up was used as the denominator. Participants contributed person-years until the date of censoring (February 1, 2013), date of first presentation with an LRE or death from any other cause.

Crude incidence was calculated for each BMI group, each SS when aged in 20s group, each SS at questionnaire completion group, and change in SS group.

#### Survival analysis

Potential confounding risk factors including self-reported comorbidities were analysed in univariate Cox proportional hazards models to determine their individual risks in liver disease.

Cox proportional hazards models were used to calculate hazard ratios (HRs) of first LRE, with 95% confidence intervals (CI). For each exposure described above, BMI, SS when aged in 20s and SS at questionnaire completion were analysed as continuous coavariates, and then BMI, SS when aged in 20s, SS at questionnaire completion and overall change in SS as categorical covariates. For each outcome, univariate models were produced. Smoking and deprivation were then added (partially adjusted), and then all covariates listed above were added, with abstinence and alcohol consumption ≥21 units/week as individual indicator variables (fully adjusted).

All analyses were performed using SPSS (version 22, SPSS Inc., Chicago, IL, USA).

## Results

### Sample characteristics

**Fig. 1:**
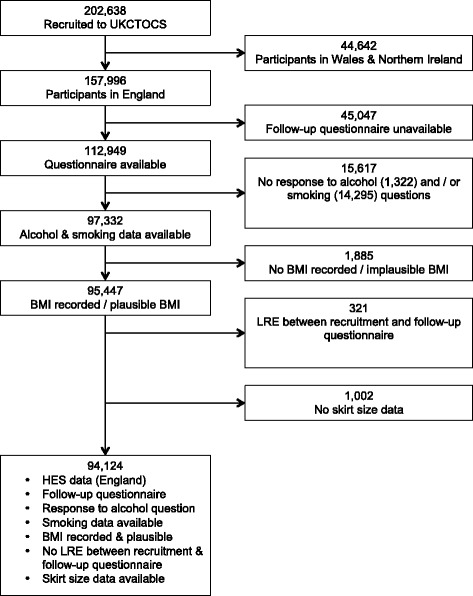
Composition of the final study cohort and its derivation from the UKCTOCS cohort

Of the 157,996 UKCTOCS participants resident in England, 62,870 were excluded including 321 women who experienced an LRE or died between recruitment and return of questionnaire and 14,295 (9%) with no data on smoking. There was some missing SS data, and the resulting effective sample size for this study was 94,124 (Fig. 1).

Overall, 97% of the participants were Caucasian, 36% were smokers, 55% were overweight (37%) or obese (18%). Median age at completion of the questionnaire was 64 years. Baseline characteristics are shown in Table 1.

**Table 1:** Baseline characteristics and number of first events according to BMI category, and for all participants

Characteristic	BMI category (kg/m^2^)			All participants
	< 25	25 - < 30	≥18	
Total, *n* (% of all participants)	42, 077 (44.7)	34,690 (36.9)	17,260 (18.3)	94,124
LRE, *n* (% of all participants)	102 (31.7)	123 (38.2)	97 (30.1)	322
Age at questionnaire return, median years (range)	63 (52–80)	64 (53–80)	64 (53–80)	64 (52–80)
IMD, mean (SD)	17.0 (13.1)	18.7 (14.1)	21.3 (15.4)	18.4 (14.0)
Smoker, *n* (%)	14,632 (34.8)	12,511 (36.1)	6548 (37.7)	33,691 (35.8)
Hypertension, *n* (%)	9382 (22.3)	11,970 (34.5)	8307 (47.9)	29,659 (31.5)
Heart disease, *n* (%)	1698 (4.0)	2052 (5.9)	1392 (8.0)	5142 (5.5)
Hypercholesterolaemia, *n* (%)	7901 (18.8)	9044 (26.1)	5369 (30.9)	22,314 (23.7)
Stroke, *n* (%)	523 (1.2)	552 (1.6)	314 (1.8)	1389 (1.5)
Diabetes, *n* (%)	827 (2.0)	1653 (4.8)	2221 (12.8)	4701 (5.0)
Rheumatoid arthritis, *n* (%)	1592 (3.8)	1742 (5.0)	1185 (6.8)	4519 (4.8)
Osteoarthritis, *n* (%)	5503 (13.1)	5822 (16.8)	4016 (23.1)	15,341 (16.3)
Osteoporosis, *n* (%)	3808 (9.1)	2082 (6.0)	770 (4.4)	6660 (7.1)
Alcohol consumption (units/week), *n* (%)				
None	8365 (19.9)	8.043 (23.2)	5432 (31.3)	21,840 (23.2)
< 1–15	31,567 (75.0)	25,095 (72.3)	11,347 (65.4)	68,009 (72.3)
16–20	1436 (3.4)	1063 (3.1)	364 (2.1)	2863 (3.0)
≥ 21	709 (1.7)	489 (1.4)	214 (1.2)	1412 (1.5)
Skirt size when aged in 20s, *n* (%)				
≤ 16	41,428 (98.5)	33,835 (97.5)	15,691 (90.4)	90,954 (96.6)
≥ 18	649 (1.5)	855 (2.5)	1666 (9.6)	3170 (3.4)
Skirt size at time of questionnaire completion, *n* (%)				
≤ 16	40,792 (96.9)	26,982 (77.8)	4481 (25.8)	72,255 (76.8)
≥ 18	1285 (3.1)	7708 (22.2)	12,876 (74.2)	21,869 (23.2)
Change in skirt size, median (interquartile range)	0.0244 (0.03)	0.0408 (0.04)	0.0667 (0.05)	0.0323 (0.04)
Overall change in skirt size, *n* (%)				
Decrease	4811 (11.4)	1153 (3.3)	362 (2.1)	6326 (6.7)
No change	12,344 (29.3)	3422 (9.9)	731 (4.2)	16,497 (17.5)
Increase	24,922 (59.2)	30,115 (86.8)	16,264 (93.7)	71,301 (75.8)

### Distributions of BMI and skirt size

**Fig. 2:**
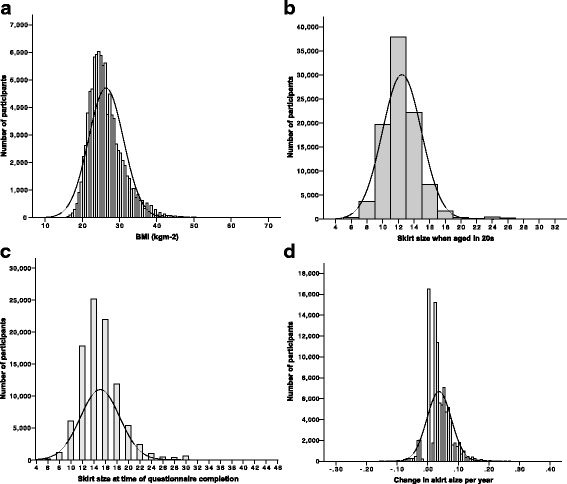
Distributions of **a**) BMI, **b**) skirt size in 20s, **c**) skirt size at questionnaire completion, and **d**) change in skirt size per year

The distributions of BMI, SS when aged in 20s, SS at questionnaire completion and annual change in SS are shown in Fig. 2. Median BMI was 25.57 kg/m^2^ (IQR 22.79–28.36), median SS when aged in 20s was 12 (IQR 10–14), median SS at questionnaire completion was 14 (IQR 12–16), and the median change in SS unit per year was 0.0323 (IQR 0.0123–0.0523). This is the equivalent to an increase of one SS unit (e.g. from 12 to 14) every 31 years.

Visual inspection of the histograms (Fig. 2), quantile-quantile plots and box plots for each outcome variable showed that each variable was approximately normally distributed, but with right-skewness seen with BMI, SS when aged in 20s and SS at questionnaire completion (BMI – skewness 1.368 (SE = 0.008), kurtosis 4.033 (SE = 0.016); SS when aged in 20s – skewness 1.442 (SE = 0.008), kurtosis 5.787 (SE = 0.016); SS at questionnaire completion – skewness 0.999 (SE = 0.008), kurtosis 2.415 (SE = 0.016); change in SS per year – skewness 0.470 (SE = 0.008), kurtosis 3.095 (SE = 0.016)).

### Crude event rates

Three hundred and twenty two (0.34%) women experienced a first LRE over the follow up period. Crude rates of LRE are shown in Table 2, categorised by BMI, SS when aged in 20s, SS at questionnaire completion and overall change in SS. The most common incident ICD-10 code was K76 (Additional file 1: Table S1).

**Table 2:** Crude rates of liver-related event. Events per 1000 participant years (95% confidence intervals)

Exposure		Event rate
BMI (kg/m^2^)	< 25	0.453 (0.369–0.550)
	25- < 30	0.661 (0.549–0.788)
	≥30	1.044 (0.847–1.273)
Skirt size in 20s	≤ 16	0.621 (0.553–0.696)
	≥ 18	1.124 (0.677–1.755)
Skirt size at questionnaire completion	≤ 16	0.550 (0.479–0.629)
	≥ 18	0.928 (0.762–1.120)
Change in skirt size/year	Decrease	0.3867 (0.206–0.661)
	No change	0.599 (0.449–0.784)
	Increase	0.669 (0.590–0.757)

**Fig. 3:**
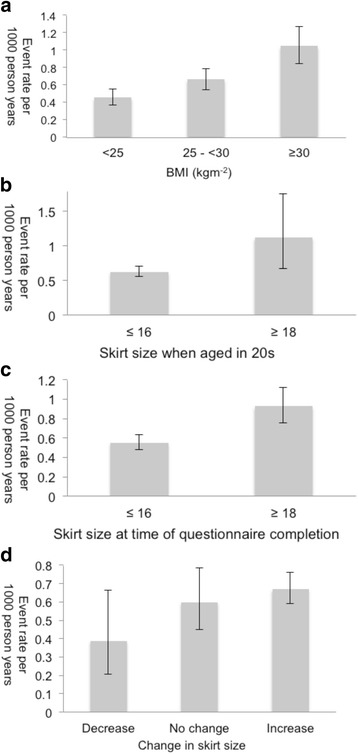
Crude rates of LRE per 1000 person years, for **a**) BMI, **b**) skirt size in 20s, **c**) skirt size at questionnaire completion and **d**) change in skirt size

The rate of LRE increased with increasing BMI. Comparison of rates of LREs in SS categories found a higher incidence in participants with SS ≥ 18, compared to participants with SS ≤ 16, both in the SS when aged in 20s group and the SS at questionnaire completion group. In terms of overall change in SS, event rate was lowest in the group where SS decreased. The rate was higher if there was no change, and highest if there was an increase in SS (Fig. 3).

### Survival analysis

#### Cox proportional model estimates for each potential confounder

There were significant associations between LRE and smoking, deprivation, BMI, heart disease, hypercholesterolaemia, diabetes, rheumatoid arthritis, alcohol abstinence and alcohol excess (≥21 units/week) (Additional file 1: Table S2). A “J-shaped” relationship between alcohol and risk of CLD is seen, and we have previously explored this finding in the UKCTOCS population [7].

#### Cox proportional model estimates for each exposure

When SS when aged in 20s ≥18 was compared to ≤16, HR for LRE was increased in the unadjusted (HR = 1.81 (95% CI; 1.14–2.87)), partially adjusted (HR = 1.68 (95% CI; 1.06–2.68)) and fully adjusted (HR = 1.39 (95% CI; 0.87–2.23)) models. The confidence interval for the fully adjusted model crossed unity, suggesting that a component of the risk may be partially attributable to one or more of the metabolic comorbidities (hypertension, hypercholesterolaemia, diabetes and heart disease) (Table 3). Comparing the two SS groups at questionnaire completion, HRs were again higher in the higher SS group in all models (HR = 1.69 (95% CI; 1.34–2.13) in the unadjusted model, HR = 1.58 (95% CI; 1.25–2.00) in the partially adjusted model, HR = 1.37 (95% CI; 1.07–1.75) in the fully adjusted model).

**Table 3:** Hazard ratios of first events for skirt size in 20s, skirt size at questionnaire completion, BMI and change in skirt size (95% confidence intervals and *p* values)

Variable				Hazard ratio (95% CI)
Skirt size when aged in 20s	Univariate	Continuous		1.062 (1.022–1.104) *p* = 0.002
		Categorical	≤ 16	Reference
			≥ 18	1.806 (1.136–2.871) *p* = 0.012
	Adjusted for smoking, deprivation		≤ 16	Reference
			≥ 18	1.681 (1.057–2.675) *p* = 0.028
	Adjusted for age, smoking, deprivation, hypertension, heart disease, hypercholesterolaemia, stroke, diabetes, rheumatoid arthritis, osteoarthritis, osteoporosis, alcohol abstinence, alcohol ≥21 units/week		≤ 16	Reference
			≥ 18	1.390 (0.868–2.226) *p* = 0.171
Skirt size at time of questionnaire completion	Univariate	Continuous		1.091 (1.062–1.121) *p* < 0.0005
		Categorical	≤ 16	Reference
			≥ 18	1.690 (1.342–2.129) *p* < 0.0005
	Adjusted for smoking, deprivation		≤ 16	Reference
			≥ 18	1.579 (1.250–1.995) *p* < 0.0005
	Adjusted for age, smoking, deprivation, hypertension, heart disease, hypercholesterolaemia, stroke, diabetes, rheumatoid arthritis, osteoarthritis, osteoporosis, alcohol abstinence, alcohol ≥21 units/week		≤ 16	Reference
			≥ 18	1.369 (1.071–1.749) *p* = 0.012
BMI (kg/m^2^)	Univariate	Continuous		1.063 (1.044–1.082) *p* < 0.0005
		Categorical	< 25	Reference
			≥ 25 - < 30	1.461 (1.123–1.899) *p* = 0.005
			≥ 30	2.308 (1.748–3.047) *p* < 0.0005
	Adjusted for smoking, deprivation		< 25	Reference
			≥ 25 - < 30	1.403 (1.076–1.830) *p* = 0.012
			≥ 30	2.162 (1.631–2.864) *p* < 0.0005
	Adjusted for age, smoking, deprivation, hypertension, heart disease, hypercholesterolaemia, stroke, diabetes, rheumatoid arthritis, osteoarthritis, osteoporosis, alcohol abstinence, alcohol ≥21 units/week		< 25	Reference
			≥ 25 - < 30	1.353 (1.034–1.770) *p* = 0.028
			≥ 30	1.880 (1.395–2.533) *p* < 0.0005
Change in skirt size/year	Univariate	Categorical	Decrease	Reference
			No change	1.554 (0.847–2.850) *p* = 0.155
			Increase	1.736 (0.994–3.031) *p* = 0.052
	Adjusted for smoking, deprivation		Decrease	Reference
			No change	1.714 (0.915–3.211) *p* = 0.092
			Increase	1.873 (1.050–3.343) *p* = 0.034
	Adjusted for age, smoking, deprivation, hypertension, heart disease, hypercholesterolaemia, stroke, diabetes, rheumatoid arthritis, osteoarthritis, osteoporosis, alcohol abstinence, alcohol ≥21 units/week		Decrease	Reference
			No change	1.781 (0.950–3.337) *p* = 0.072
			Increase	1.799 (1.007–3.214) *p* = 0.047

Compared to women whose SS decreased between their 20s and questionnaire completion, HRs were higher in those whose SS did not change and highest in those whose SS increased (Table 3).

Compared to normal BMI, overweight and obesity were significantly associated with LRE in all models (Table 3).

## Discussion

### Main findings

We have demonstrated in a cohort of post-menopausal women that a larger SS is associated with subsequent risk of LRE, and a SS of ≥18 compared to a SS of ≤16 is associated with a higher HR than that associated with overweight, but less than that associated with obesity when compared to a normal BMI. Although the risks of high SS and high BMI may not be directly comparable, the value of communicating public health messages in terms of SS lies in better understanding amongst the general public compared to communicating the risk of liver disease associated with increased BMI.

In our cohort, 76% reported an increase in SS between when aged in 20s and questionnaire completion. This is consistent with previous studies reporting the change in body composition associated with transitioning from pre-menopausal to post-menopausal status, with an increase in central adiposity manifested by increased waist circumference (WC) [19].

When BMI and SS (as continuous variables) were combined, the HR for each was reduced, suggesting that SS (and BMI) is an independent predictor for NAFLD, and that SS may reflect centripetal fat distribution associated with NAFLD better than BMI.

NAFLD is poorly identified in primary care and it is conceivable that a proportion of individuals with LREs that were not associated with an ICD-10 code for fatty liver may have had NAFLD. SS may be a better predictor of NAFLD (obesity) related liver disease than a clinical diagnosis of NAFLD in primary care.

Although the codes or text contributing most commonly to LRE were those representing NAFLD, those representing alcoholic liver disease contributed to nearly 10% of LREs (Additional file 1: Table S1). Regardless of the aetiology of CLD, the clinicopathological pathway is progressive fibrosis leading to cirrhosis [20] and there may be common pathways in which alcohol and BMI damage the liver [21]. Patterns of alcohol consumption in women are changing; 16% of women in England consume above recommended limits, and this practice is highest in the 55–64 year old group [22], and the rate of alcohol-related hospital admissions by women increased by over 30% between 2008 and 2015 [23].

### Strengths and limitations

Strengths of this study include the large size of the cohort, the prospective design and the independence of data capture for outcomes. We used ICD-10 codes for cirrhosis that have been used in other studies, but in an attempt to maximise the ability to identify liver disease we also included codes relating to clinical consequences of advanced cirrhosis, the events defining decompensated liver disease. Evaluation of numerous possible confounders including self reported known comorbidities and socioeconomic status minimised bias.

Limitations include the reliance of self-reporting of SS, height and weight and co-morbidities. There is some evidence supporting the reliability of self-reporting of biometric data including height and weight [24–28], notably in a longitudinal study of older people [29]. There was a 30–50 year recall of participants’ SS when aged in their 20s, raising the possibility of recall error. Several studies have demonstrated good accuracy in recalled weight, with some data indicating underestimation in those with higher BMI [30–33]. We postulate that participants may have a better recollection of their skirt size than their weight or waist size. There was a 25 year age range in participants, and older participants may have had children at a younger age than younger participants, which may have increased their SS [34].

It is likely that there will be some variability between SS over the period between the two SS estimates. In the UK there is no requirement for manufacturers to adhere to the standard sizing. In addition the phenomenon of vanity sizing is recognised, where clothes with the same size label have become larger over recent decades. This has become a common practice of clothing manufacturers, which may potentially impede comparisons of sizes over time [35]. Indeed, the Chief Medical Officer for England has highlighted this ‘size inflation’ as a risk for society normalising overweight [36].

Reliance on ICD-10 to define events may result in errors due to mis-coding. We used three independent sources in an attempt to reduce risk of non-coding. Further, HES data may not capture some areas of healthcare, including the private sector. Finally, although attempts were made to ensure UKCTOCS was representative of the general population, there was a ‘healthy volunteer effect’ on overall and cause-specific mortality, which may affect the generalisability of our findings [17].

### Other studies

The link between obesity and the risk of NAFLD is strong, with a clear dose-response relationship demonstrated in cross-sectional studies [37], although data from prospective studies are limited [38, 39].

However, few studies have investigated the relationship between SS and disease. Ours is the only study we are aware of that has investigated the association between SS and liver disease. The UKCTOCS group demonstrated an increase in risk of breast cancer with increase in SS over time [12].

A study nested in the Netherlands Cohort Study on Diet and Cancer reported increased risk of endometrial cancer with increasing SS. The correlation between self-reported SS and self-reported WC, self-reported hip circumferences and BMI based on self-reported height and weight in 1334 women, were 0.71, 0.78 and 0.76 respectively [40].

A study of 293 men and women found that professionally measured WC correlated closely with clothing size in both men and women (*r* = 0.80 and 0.78, respectively) [41].

Similarly, a study nested in the fourth Glasgow monitoring cardiovascular (MONICA) disease risk factor survey measured height, weight, WC and hip circumference, and obtained SS in 161 women. Dress size correlated with WC and BMI. Dress size ≥18 was associated with a significantly increased risk of cardiovascular disease [14].

## Conclusion

We have demonstrated that SS in middle age is associated with increased risk of CLD. In post-menopausal women who develop liver disease, there is a significantly higher average SS when aged in their 20s (and in middle age). If these results are confirmed in further population studies, this may provide a simple way for women to stratify their risk of liver disease.

## Additional file

Additional file 1: **Table S1.** ICD-10 codes and death certificate text of first LREs. Summary of the ICD-10 code(s) representing first presentation of liver-related event. **Table S2.** Hazard ratios for liver-related events for potential confounders (95% confidence intervals and *p* values). Univariate hazard ratios for liver-related events for smoking, deprivation, alcohol categories, alcohol ≥21 units/week, abstinence from alcohol, BMI, hypertension, heart disease, hypercholesterolaemia, stroke, diabetes, rheumatoid arthritis, osteoarthritis, osteoporosis. (DOCX 95 kb)

## Abbreviations

BMI: Body mass index
CLD: Chronic liver disease
HES: Hospital episode statistics
HR: Hazard ratio
ICD-10: International classification of disease 10th revision
IMD: Index of multiple deprivation
IQR: Interquartile range
LRE: Liver-related event
NAFLD: Non-alcoholic fatty liver disease
NHS: National Health Service
SE: Standard error
SS: Skirt size
UKCTOCS: United Kingdom Collaborative Trial of Ovarian Cancer Screening
WC: Waist circumference
