# Supplementary material for: Association between skirt size and chronic liver disease in post-menopausal women: a prospective cohort study within the United Kingdom Trial of Ovarian Cancer Screening (UKCTOCS)
Source: BMC Public Health. 2018 Mar 27;18:409. doi: 10.1186/s12889-018-5308-x (PMC5870222; doi:10.1186/s12889-018-5308-x)
Supplement: Supplementary file 1 — Table S1. ICD-10 codes and death certificate text of first LREs. Summary of the ICD-10 code(s) representing first presentation of liver-related event. Table S2. Hazard ratios for liver-related events for potential confounders (95% confidence intervals and p values). Univariate hazard ratios for liver-related events for smoking, deprivation, alcohol categories, alcohol ≥21 units/week, abstinence from alcohol, BMI, hypertension, heart disease, hypercholesterolaemia, stroke, diabetes, rheumatoid arthritis, osteoarthritis, osteoporosis. (DOCX 95 kb) [file 12889_2018_5308_MOESM1_ESM.docx]

**ASSOCIATION BETWEEN SKIRT SIZE AND CHRONIC LIVER DISEASE IN POST-MENOPAUSAL WOMEN: A PROSPECTIVE COHORT STUDY WITHIN THE UNITED KINGDOM TRIAL OF OVARIAN CANCER SCREENING (UKCTOCS)**

**ADDITIONAL FILE**

**Table S1.** ICD-10 codes and death certificate text of first LREs

The number of codes / death certificate text results is higher than the number of LREs (322) as some participants had more than one code when presenting with first LRE

| **Source** | **Code or text** | **Number of participants (% of those with LRE)** |
| --- | --- | --- |
| Hospital admission | K70 | 15 (4.7) |
|  | K73 | 9 (2.8) |
|  | K74 | 45 (14.0) |
|  | K76 | 180 (56.9) |
|  | C22.0 | 6 (1.9) |
|  | I85 | 12 (3.7) |
|  | Z94.4 | 33 (10.2) |
| Outpatient appointment | K74 | 1 (0.3) |
|  | Z94.4 | 11 (3.4) |
| Cancer registration | C22.0 | 12 (3.7) |
| Death certificate | K70 | 6 (1.9) |
|  | K74 | 7 (2.2) |
|  | K76 | 10 (3.1) |
|  | C22.0 | 2 (0.6) |
|  | Mention of alcoholic liver disease | 8 (2.5) |
|  | Mention of non-alcoholic fatty liver disease | 8 (2.5) |

**Table S2.** Hazard ratios for liver-related events for potential confounders (95% confidence intervals and p values)

| Variable | | Hazard ratio |
| --- | --- | --- |
| Smoking | No | Reference |
|  | Yes | 1.847  (1.484-2.299)  p<0.0005 |
| IMD | Continuous | 1.019  (1.013-1.026)  p<0.0005 |
| Alcohol | None | Reference |
|  | <1-15 units/week | 0.637  (0.501-0.809)  p<0.0005 |
|  | 16-20 units/week | 0.819  (0.440-1.527)  p=0.531 |
|  | ≥21 units/week | 1.661  (0.891-3.095)  p=0.110 |
| Alcohol ≥21 units/week | No | Reference |
|  | Yes | 2.283  (1.251-4.166)  p=0.007 |
| Abstinence from alcohol | No | Reference |
|  | Yes | 1.505  (1.189-1.906) |
| BMI | Continuous | 1.063  (1.044-1.082)  p<0.0005 |
| BMI | <25 | Reference |
|  | 25-<30 | 1.461  (1.123-1.899)  p=0.005 |
|  | ≥30 | 2.308  (1.748-3.047)  p<0.0005 |
| Hypertension | No | Reference |
|  | Yes | 1.391  (1.112-1.740)  p=0.004 |
| Heart disease | No | Reference |
|  | Yes | 2.201  (1.556-3.112)  p<0.0005 |
| Hypercholesterolaemia | No | Reference |
|  | Yes | 1.679  (1.334-2.114)  p<0.0005 |
| Stroke | No | Reference |
|  | Yes | 1.722  (0.854-3.474)  p=0.129 |
| Diabetes | No | Reference |
|  | Yes | 2.810  (2.025-3.899)  p<0.0005 |
| Rheumatoid arthritis | No | Reference |
|  | Yes | 1.815  (1.224-2.692)  p=0.003 |
| Osteoarthritis | No | Reference |
|  | Yes | 1.328  (1.013-1.741)  p=0.040 |
| Osteoporosis | No | Reference |
|  | Yes | 1.784  (1.272-2.503)  p=0.001 |
